# A Multi-Criteria Decision-Making Model for Evaluating Senior Daycare Center Locations

**DOI:** 10.3390/ijerph16245031

**Published:** 2019-12-10

**Authors:** Amy H. I. Lee, He-Yau Kang

**Affiliations:** 1 Department of Technology Management, Chung Hua University, Hsinchu 300, Taiwan; amylee@chu.edu.tw; 2 Department of Industrial Engineering and Management, National Chin-Yi University of Technology, Taichung 411, Taiwan

**Keywords:** analytic network process (ANP), interpretive structural modeling (ISM), fuzzy, senior daycare center, location

## Abstract

Many developed and developing countries are facing an imminent population aging and rapid demographics changing problem. The need of various kinds of eldercare is increasing tremendously. A senior daycare center, very similar to a daycare center for toddlers and preschoolers, can provide the elderly a place to go during daytime and have a more diversified social life. In this research, a senior daycare center location evaluation problem is studied, and a model for facilitating the decision-making of the senior daycare center location is constructed by considering the benefits, opportunities, costs, and risks (BOCR) of the locations. Senior daycare center location evaluation factors are listed first through literature review and interview with experts. These factors are used to construct a network, which is applied to prepare a questionnaire to ask about the influences of a criterion to other criteria. The interrelationships among the criteria are calculated by adopting fuzzy interpretative structural modeling (FISM). Based on the results from the FISM, a fuzzy analytic network process (FANP) questionnaire is given out, and the results are used to determine the priorities of the criteria. In addition, the final ranking of the senior daycare center locations can be obtained. The research results can provide references for prospective senior daycare center providers for making relevant decisions.

## 1. Introduction

In many developed countries and developing countries, the fertility rate keeps declining, and life expectancy is increasing. As family size decreases and employment rate among women increases, the traditional way of family caregiving is declining, and thus the demand for long-term care services is increasing. Long-term care service thus has become very critical in today’s society. Some common long-term care service options are: home and community care, palliative or hospice care, board and care homes, assisted living (non-medical senior housing), subsidized senior housing (non-medical), and continuing care retirement communities (CCRCS) [1]. Policies aims to enable “aging in place” are present in many countries that encourage older people to remain in their community to face the challenge of an aging population [2,3]. Societal alienation experienced by seniors is correlated with health risks and negative well-being, and thus, increasing the support and empowerment of seniors so that they can proactively improve the quality of life for themselves and for other seniors around them is recommended [4]. Senior daycare center is a kind of home and community care, and the services provided may involve nurses, physical therapists, speech therapists, occupational therapists, and social workers [1]. Thus, seniors can engage in social and therapeutic activities and receive mental stimulation. The quality of life of seniors and their families can be enhanced as a result.

Although some governments have been implementing long-term care plans, the long-term care needs are usually rested on the families, which are often nuclear-centered, or even the elderly themselves. Therefore, the selection of the most suitable long-term care service based on the situation of care recipients and their family members is becoming an important decision. On the other hand, long-term care institutions need to provide good quality services to improve the living quality of elderly and to decrease the load of their families. When starting a new long-term care service, there are many aspects of service development that need to be considered, and the selection of the service location is an essential decision.

With the swift demographic and societal changes, Taiwan needs to face the aging problem seriously, and one direction is to provide good-quality eldercare services. Although the government has implemented some measures for the long-term care in Taiwan, most of the cares are designated for underprivileged citizens. As a result, the majority of seniors and their families need to be financially responsible for receiving the services. With the tradition of elderly living with their families and the family members being responsible for the elderly, senior daycare center may be a good option. The elderly can be taken care of during the daytime and can have their own social lives while the family members can go to work and school without worrying about the elderly being left alone at home. With population aging, the demand of senior daycare centers has been increasing consequently. Most of these centers are privately operated, and finding an appropriate location is the first task faced by service providers when setting up a senior daycare center.

The purpose of this research is to focus on senior daycare centers and to construct a multi-criteria decision-making model to prospective service providers for evaluating senior daycare center locations. The benefits, opportunities, costs, and risks (BOCR) merits of locations are considered to develop a network for selecting the most appropriate location for setting up a senior daycare center. Important criteria are listed under each merit, and the interrelationships among the criteria under each merit are analyzed by the fuzzy interpretative structural modeling (FISM). The results are used to develop a network, and the fuzzy analytic network process (FANP) is adopted to calculate the weights of the criteria and to obtain the ranking of the location alternatives. The proposed model can thus facilitate the senior daycare center providers in selecting the most appropriate location for developing senior daycare centers.

The rest of the paper is organized as follows. Some recent works that studied the long-term care services and policies in different countries are reviewed in Section 2. The demography and long-term care in Taiwan are briefly introduced in Section 3. A multi-criteria decision-making model for evaluating senior daycare center locations is constructed in Section 4. Section 5 presents a case study. Section 6 contains the conclusions and future research directions.

## 2. Current Long-Term Care Services

As the population ages with the increasing life expectancy, the cost of long-term care is becoming a skyrocketing burden in the health and social care systems in many countries, and the cost constitutes an ever-larger proportion of public budgets [5]. Some countries, especially developed countries, have financed and developed long-term care programs, and many countries are developing or are planning to develop related programs. For instance, the Singapore government has emphasized that the family should be responsible for the elderly to show filial piety, and the government has relied primarily on voluntary welfare organizations and some on private, for-profit long-term care services [6,7]. In addition, the financing of long-term care relies on a responsibility-shared healthcare system and targets at the lower-income groups. Rozario and Rosetti [6] analyzed long-term care policies, programs, and practices of aging in Singapore from the political economy perspective. Some specific long-term care policy areas, from home-based, community-based, to institutional-based programs, were introduced. Won [8] presented the national mandatory elderly long-term care insurance in Korea and suggested to build an efficient linkage between long-term care and health care, which were two separate systems in Korea. Rhee et al. [9] examined three countries, namely South Korea, Japan, and Germany, about how they used social insurance to finance medical care and their current long-term care insurance systems. The authors contrasted the financing of the systems in the three countries and made some suggestions regarding revenue generation, benefits design, and eligibility. Pin and Spini [10] focused on the very old population, which is rising with the increase of life expectancy in most industrialized and developing countries, and highlighted some major challenges for the caring of the very old adults. The authors then proposed some approaches for policies and programs to meet the complex care need of the very old adults and their families, to balance between formal and informal care, and to develop suitable places for living. Chin and Phua [7] reviewed the long-term care service in Singapore. The authors presented some challenges, such as the perverse financial incentives for hospitalization, the over-reliance on voluntary welfare organizations, and the quality of informal family caregivers.

Even though some kind of public long-term care insurance program may have been provided in some countries, not every citizen may be covered, and the support may not be adequate. Many countries encourage individuals to purchase private long-term care insurance to cover their support needs. Therefore, there is a growing demand of voluntary private long-term care insurance so that individuals and families can protect themselves against the financial risk posed by long term care needs [5]. Strier and Werner [11] introduced the current condition of a community-based social long-term care insurance program in Israel, and how the program achieved some multiple goals and improved the living condition of older people. The authors also provided reasons why people with Alzheimer’s disease (AD) and other types of dementia or their relatives declined to make use of the services. Nadash and Cuellar [5] discussed how Germany, which already had public long-term care insurance program, promoted the public to purchase supplemental private long-term care insurance by subsidizing the purchase of qualified policies. The authors provided data on private insurance market expansions and the extent of the policy goals being achieved.

## 3. Demography and Long-Term Care in Taiwan

Taiwan, like many other developing and developed countries in the world, is facing the challenge of a rapidly aging population. The rapid demographic change is due to rising life expectancies and declining fertility rates. With medical advancement and good healthcare system, it is expected that the average life expectancy will reach 89.0 years for women and 82.2 years for men in Taiwan by 2056 [12]. In 1993, Taiwan entered the aging society, which is defined by the United Nations for populations with more than 7% elderly at aged 65 or higher. In Taiwan, the elderly population rises every year. Taiwan has entered the aged society, defined for populations with more than 14% elderly at aged 65 or higher, in 2018. Taiwan is expected to enter the super-aged society, defined for populations with more than 20% elderly, in 2026 [13]. It is expected that the percentage of Taiwan’s population classified as elderly would increase from 12% in 2015 to 42% in 2060 [14]. In fact, in February, 2017, the percentage of elderly (65+ in age) exceeded the percentage of youth (0–14 in age), and the aging index, which is defined as the population of elderly divided by the population of youth, was 100.18 [13]. It is expected that Taiwan will be the second fast aging society transition into an aged society, with only 25 years, only after Japan which had the transition with 24 years [14]. Elderly dependency ratio is a ratio of people above working age (65+) to workforce of a country, and child dependency ratio is a ratio of people aged 0–14 to workforce of a country. In Taiwan, the total dependency ratio reached the lowest point of 34.7% in 2012 and started to increase ever since. The total dependent population that every 100 persons of working age needed to support was 36.2 people in 2016, with 18.2 children and 18.0 elderly [15]. The family structure in Taiwan has changed over the last five decades with a decline in family size and an increase in nuclear families. By the end of 2010, the average number of persons per household in Taiwan was 3.0 [16]. The living arrangement of the elderly has been changing too. In 2013, the percentage of the elderly who lived alone was 11.1%, who lived only with spouse/partner was 20.6%, lived with children and other family members was 64.9%, and others was 3.4% [17].

The government in Taiwan implemented a ten-year long-term care plan starting 2007 to develop an aging-in-place network. Under the implementation of the plan, the total institutions that provided various kinds of services and the total people who received different care services increased. Nevertheless, the plan encountered numerous problems, and a 10-year long-term care 2.0 plan was implemented starting from 2017. The objective of the new long-term care plan is to create localized, community-based long-term care model that integrates social care, medical care, and preventive health resources [18]. The number of services provided by the long-term care 2.0 plan increased from eight to a total of 17 items. The new categories included dementia care, family-care support, community-based preventive care, integrated services for aboriginal groups in remote areas, and hospital discharge plans and transition care [14]. It is expected that the number of care recipients will increase by 44% from approximately 511,000 to about 738,000 [18].

The long-term care condition in Taiwan has been studied. Chen [12] discussed the challenges of eldercare in Taiwan’s aging society. The main challenges included: the need to build a comprehensive eldercare system, inadequacy in home and community care, diminishing dwindling function of family care, heavy reliance on foreign caregivers, special care needs of elderly, overutilization of medical resources, and the problem of polypharmacy. Chen et al. [19] briefly introduced the challenges in population aging in Taiwan, the urban aging in Japan, and the elderly long-term care insurance in Korea. The population aging and governmental strategies for five major metropolitan areas in Taiwan, namely, Taipei City, New Taipei City, Taichung City, Tainan City, and Kaohsiung City, were presented. Lin et al. [20] defines the long-term aging health care system based on five dimensions and twenty criteria. A DANP-mV model, which integrates the decision-making trial and evaluation laboratory (DEMATEL), ANP and a modified VIKOR, was proposed to analyze the current system in Taiwan and to develop a strategy for continuous improvement.

## 4. A Senior Daycare Center Location Evaluation Model

A senior daycare center location evaluation model is developed by incorporating the FISM and the FANP. Fuzzy set theory is incorporated in the model because uncertainty and ambiguity is often present in decision-making and in the real business environment. For instance, decision-makers may not have complete information or a full understanding of all aspects of the problem, and the experiences and judgments of humans are not well-defined [21]. The adoption of the fuzzy set theory thus can help encounter the problem. The ANP, a generalization of the analytic hierarchy process [22], can be applied to solve a problem with dependence and feedback [23,24]. The ANP with BOCR is a type of the ANP, and the fuzzy ANP with BOCR has been applied in solving different kinds of problems, for example, supplier selection [25], production strategy evaluation [26], and technology transfer of new equipment [27]. The ISM can be adopted to analyze the interrelationships in a network, and the ISM incorporated with the fuzzy set theory has also been used in solving different problems, such as technology selection [28], wind farm performance evaluation [29], new product development [30], and vendor selection [31].

**Figure 1:**
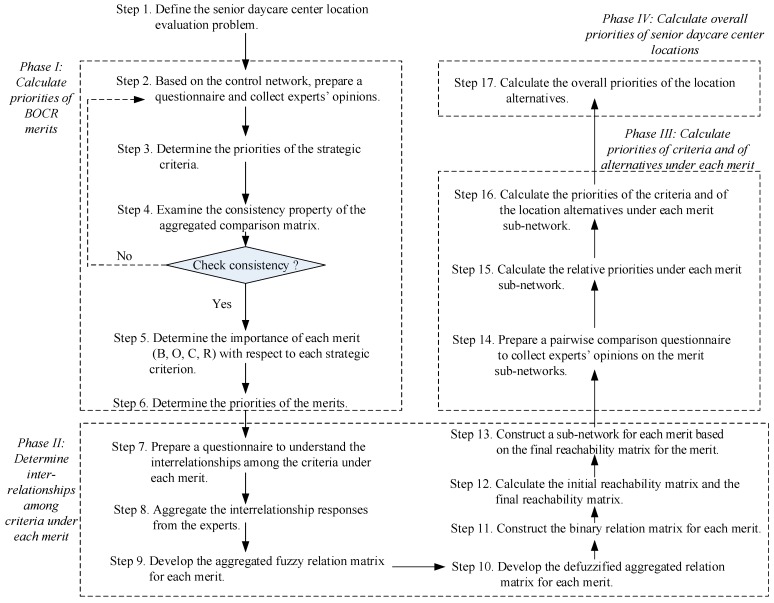
The senior daycare center evaluation model.

In the proposed model, the FISM can be applied to determine the interrelationships among the criteria and among the sub-criteria. The FANP, based on the results from the FISM, can be adopted to evaluate the priorities of the criteria, and the ranking of the senior daycare center locations. The procedures of the model are depicted in Figure 1 and are as follows:

Step 1. Define the senior daycare center location evaluation problem and decompose the problem into a network with a control network and four BOCR sub-networks. Perform a comprehensive review of past works related to long-term care services and location selection models, and interview with experts in the long-term care service field. An initial network with a control network and four BOCR sub-networks can be developed after the confirmation of the experts.

*Phase I: Calculate priorities of BOCR merits* [23,25,26]

Step 2. Based on the control network, prepare a questionnaire and collect experts’ opinions. Under the control network, there are strategic criteria for attaining the goal of selecting the most appropriate senior daycare center. Experts are invited to pairwise compare the importance of the strategic criteria.

Step 3. Calculate the priorities of the strategic criteria. Transform the pairwise comparison results from each expert in the questionnaire into triangular fuzzy numbers using Table 1 [27]. Use the geometric mean approach to aggregate the opinions of the experts, and develop a fuzzy-aggregated pairwise comparison matrix. The centroid method is adopted to defuzzify the fuzzy numbers in the fuzzy-aggregated pairwise comparison matrix, and a defuzzified-aggregated pairwise comparison matrix is obtained. Calculate the maximum eigenvalue and the eigenvector of the defuzzified-aggregated pairwise comparison matrix [22]:

(1) $$A\cdot w=\lambda_{max}\cdot w,$$

where A is the defuzzified-aggregated pairwise comparison matrix, w is the eigenvector, and $\lambda_{\max}$ is the largest eigenvalue of A.

Step 4. Examine the consistency property of the defuzzified-aggregated comparison matrix. The consistency of the defuzzified aggregated pairwise comparison matrix is examined by calculating the consistency index (CI) and the consistency ratio (CR) [22]:

(2) $$CI=\frac{\lambda_{max}-q}{q-1},$$

(3) CR=CI/RI,

where *q* is the number of strategic criteria, and RI is the random index [22]. If the consistency test of a matrix fails, i.e., CR exceeds 0.1, the experts need to revise the questionnaire.

Step 5. Determine the importance of each merit (B, O, C, R) with respect to each strategic criterion. Experts are invited to rate the importance of each merit with respect to each strategic criterion using a five-level scale. The linguistic term and the triangular fuzzy number of each scale are: very high (7,9,9), high (5,7,9), medium (3,5,7), low (1,3,5), and very low (1,1,3). Experts’ opinions are aggregated by the geometric mean approach, and the centroid method is applied to defuzzify the fuzzy numbers. The defuzzified weights of the strategic criteria are normalized.

Step 6. Calculate the priorities of the four merits. The priority of a merit is obtained by multiplying the priority of a merit on each strategic criterion from Step 5 with the priority of the respective strategic criterion from Step 3 and summing up all the calculated values for the merit. The normalized priorities of benefits (B), opportunities (O), costs (C) and risks (R) merits are b, o, c, and r, respectively.

*Phase II: Determine interrelationships among criteria under each merit* [28,31,32]

Step 7. Prepare a questionnaire to understand the interrelationships among the criteria under each merit. Experts are asked to determine the interrelationships between each two criteria under each merit. For example, the relationship between each two criteria can be from *x_i_* to *x_j_*, from *x_j_* to *x_i_*, both from *x_i_* to *x_j_* and from *x_j_* to *x_i_*, or no relationship between *x_i_* and *x_j_*. Five levels of relationship are set, and the linguistic variables are transformed into triangular fuzzy numbers, as shown in Table 2 [28,30].

Step 8. Aggregate the interrelationship responses from the experts. The geometric mean approach is used to aggregate experts’ opinions. The relation from criterion *x_i_* to criterion *x_j_* is represented by a triangular fuzzy number, ${\tilde{x}}_{ij}$.

Step 9. Develop the aggregated fuzzy relation matrix for each merit. An aggregated fuzzy relation matrix can be formed by adopting α -cuts. The fuzzy relation matrix for the criteria under a merit is [32]:

(4) $$D_{\chi }^{\alpha }=\left[\begin{array}{ccccc} 0 & \left[x_{12L}^{\alpha }\;,\;x_{12U}^{\alpha }\right] & \cdots & \cdots & \left[x_{1nL}^{\alpha }\;,\;x_{1nU}^{\alpha }\right]\\ \left[x_{21L}^{\alpha }\;,x_{21U}^{\alpha }\right] & 0 & \cdots & \cdots & \left[x_{2nL}^{\alpha }\;,x_{2nU}^{\alpha }\right]\\ \vdots & \vdots & 0 & \left[x_{ijL}^{\alpha }\;,\;x_{ijU}^{\alpha }\right] & \vdots \\ \vdots & \vdots & \left[x_{jiL}^{\alpha }\;,\;x_{jiU}^{\alpha }\right] & \ddots & \vdots \\ \left[x_{n1L}^{\alpha }\;,\;x_{n1U}^{\alpha }\right] & \left[x_{n2L}^{\alpha }\;,\;x_{n2U}^{\alpha }\right] & \cdots & \cdots & 0 \end{array}\right],$$

where 0≤α≤1, χ= B,O,C,R.

Step 10. Develop the defuzzified-aggregated relation matrix for each merit. By adopting α -cuts and the degree of satisfaction of the experts on judgment, $X_{\alpha }^{\beta }$, the defuzzified-aggregated relation matrix can be formed. If α is fixed, the index of optimism β can be set to represent the degree of the optimism of experts [32]. A larger β represents a higher degree of optimism, and vice versa. The index of optimism is a linear convex combination. The relation from criterion *i* to criterion *j*, with α and β, is:

(5) $$x_{ij}^{\alpha \beta }=(1-\beta )x_{ijL}^{\alpha }+\beta x_{ijU}^{\alpha },\;\forall \beta \in \left[0,1\right],$$

The defuzzified-aggregated relation matrix for merit χ, $D_{\chi }^{\alpha \beta }$, is:

(6) $$D_{\chi }^{\alpha \beta }=\left[\begin{array}{ccccc} 0 & x_{12}^{\alpha \beta } & \cdots & \cdots & x_{1n}^{\alpha \beta }\\ x_{21}^{\alpha \beta } & 0 & \cdots & \cdots & x_{2n}^{\alpha \beta }\\ \vdots & \vdots & 0 & x_{ij}^{\alpha \beta } & \vdots \\ \vdots & \vdots & x_{ji}^{\alpha \beta } & \ddots & \vdots \\ x_{n1}^{\alpha \beta } & x_{n2}^{\alpha \beta } & \cdots & \cdots & 0 \end{array}\right]$$

Step 11. Construct the binary relation matrix for each merit. A threshold value, ξ is set to determine whether there is a significant relation between the two criteria. If $x_{ij}^{\alpha \beta }$ is higher than the threshold value, *x_j_* is deemed reachable from *x_i_*, and let $\pi_{ij}=1$, otherwise, $\pi_{ij}=0$ [31]. A binary relation matrix $D_{\chi }$ is generated.

If $x_{ij}^{\alpha \beta }\geq \xi$ then $\pi_{ij}=1$; otherwise, $\pi_{ij}=0$ for all *i,j.*

(7) $$D_{\chi }^{}=\left[\begin{array}{ccccc} 0 & \pi_{12}^{} & \cdots & \cdots & \pi_{1n}^{}\\ \pi_{21}^{} & 0 & \cdots & \cdots & \pi_{2n}^{}\\ \vdots & \vdots & 0 & \pi_{ij}^{} & \vdots \\ \vdots & \vdots & \pi_{ji}^{} & \ddots & \vdots \\ \pi_{n1}^{} & \pi_{n2}^{} & \cdots & \cdots & 0 \end{array}\right]$$

Step 12. Calculate the initial reachability matrix and the final reachability matrix. Use criteria as an example. By adding $D_{\chi }$ and the identity matrix **I**, an initial reachability matrix $H_{\chi }$ can be calculated. For the criteria, by raising $H_{\chi }$ to powers using the Boolean multiplication and addition, the final reachability matrix, ${H_{\chi }}^{*}$, can be obtained where convergence is met and the transitivity of the contextual relation among factors is achieved [28].

(8) $$H_{\chi }=D_{\chi }+I,$$

(9) $${H_{\chi }}^{*}={H_{\chi }}^{k}={H_{\chi }}^{k}{}^{+1},\;k>\;1,$$

Step 13. Construct a sub-network for each merit based on the final reachability matrix for the merit.

*Phase III: Calculate priorities of criteria and of alternatives under each merit* [23,29]

Step 14. Prepare a pairwise comparison questionnaire to collect experts’ opinions on the merit sub-networks. A questionnaire is formulated based on the sub-networks constructed in Step 13 to pairwise compare the importance of the criteria under each merit, the interdependence among the criteria under each merit, and the expected performance of the senior daycare center location alternatives with respect to each criterion. The nine different linguistic terms in Table 1 are used.

Step 15. Calculate the relative priorities under each merit sub-network. A similar procedure as in Steps 3 and 4 is used to calculate the relative importance weights of the criteria with respect to the same upper-level merit, the interdependence of the criteria with respect to the same upper-level merit, and the expected relative performance of location alternatives with respect to each criterion.

**Figure 2:**
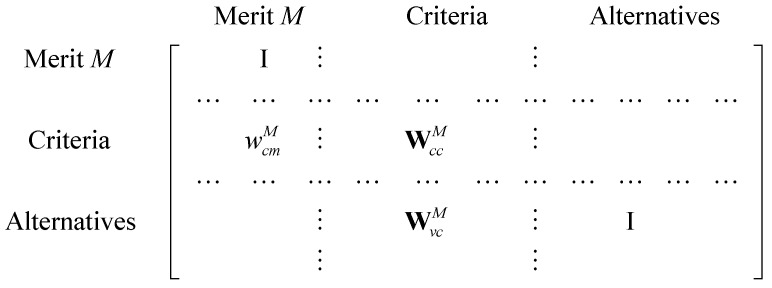
Unweighted supermatrix for merit *M*.

Step 16. Calculate the priorities of the criteria and of the location alternatives under each merit sub-network. Using the priorities obtained from Step 15, form an unweighted supermatrix for merit *M*, as depicted in Figure 2, where $w_{cm}^{M}$ is a vector that represents the impact of merit *M* on the criteria, $W_{cc}^{M}$ indicates the interdependency of the criteria under merit *M*, $W_{vc}^{M}$ is a matrix that represents the impact of criteria on each of the location alternatives under merit *M*, and I is the identity matrix [29]. A weighted supermatrix and a limit supermatrix for each sub-network can be calculated by ANP, which is proposed by Saaty [23]. The priorities of the alternatives under each merit are shown in the alternative-to-merit column of the limit supermatrix for the merit. The priorities of the criteria under each merit sub-network can be obtained by forming a similar unweighted supermatrix in Figure 2, and only merit *M* and criteria information is included. After calculating the weighted supermatrix and the limit supermatrix, the priorities of criteria under each merit are shown in the criteria-to-merit column of the limit supermatrix for the merit.

*Phase IV: Calculate overall priorities of senior daycare center locations* [24,25]

Step 17. Calculate the overall priorities of the location alternatives. The overall priority of a location alternative can be obtained by synthesizing the priorities of the location alternative under each merit from Step 16 with the corresponding normalized weights *b*, *o*, *c,* and *r* from Step 6. Six different ways can be used to calculate the overall priority of each location alternative [24,25,33,34,35,36]. The bipolar method is developed based on Tchangani [33], Tchangani [34], Tchangani [35], and Tchangani et al. [36].

1. Additive:

(10) $$P_{v}^{}=bB_{v}^{}+oO_{v}^{}+c\left[{\left(1/C_{v}\right)}_{\mathrm{Normalized}}\right]+r\left[{\left(1/R_{v}\right)}_{\mathrm{Normalized}}\right],$$

where *B_v_*, *O_v_*, *C_v_*, and *R_v_* represent respectively the synthesized results of alternative *v* under merit B, O, C, and R, and *b*, *o*, *c,* and *r* are respectively the normalized weights of merit B, O, C, and R.

2. Probabilistic additive:

(11) $$P_{v}^{}=bB_{v}^{}+oO_{v}^{}+c(1-C_{v})+r(1-R_{v}),$$

3. Subtractive:

(12) $$P_{v}^{}=bB_{v}^{}+oO_{v}^{}-cC_{v}-rR_{v},$$

4. Multiplicative priority powers:

(13) $$P_{v}^{}=B_{v}^{b}O_{v}^{o}{\left[{\left(1/C_{v}\right)}_{\mathrm{Normalized}}\right]}^{c}{\left[{\left(1/R_{v}\right)}_{\mathrm{Normalized}}\right]}^{r},$$

5. Multiplicative:

(14) $$P_{v}^{}=B_{v}O_{v}/C_{v}R_{v},$$

6. Bipolar:

(15) $$\mathrm{Selectability}:\;\;\;\;P_{v}^{+}=\frac{b}{\left(b+o\right)}B_{v}+\frac{o}{\left(b+o\right)}O_{v},$$

(16) $$\mathrm{Rejectability}:\;\;\;\;P_{v}^{-}=\frac{c}{\left(c+r\right)}C_{v}+\frac{r}{\left(c+r\right)}R_{v}.$$

A case study of a senior daycare center location evaluation problem is presented next, to examine the practicality of the proposed model. The results will provide a comprehensive framework and guidance to practitioners in evaluating the expected performance of different daycare center locations.

## 5. Case Study

**Figure 3:**
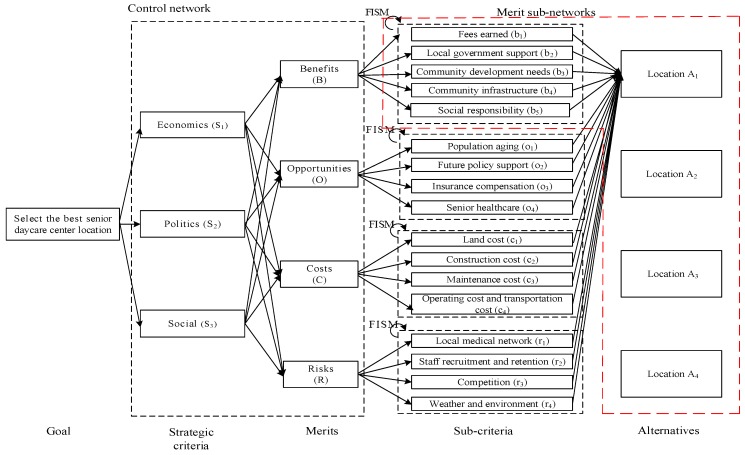
Senior daycare center location evaluation network.

Based on the proposed senior daycare center location evaluation model, a case study is carried out in Taiwan. A comprehensive literature review on evaluating long-term care services is performed, and experts in the field are asked for their opinions on evaluating senior daycare centers locations. Because the majority of long-term care services in Taiwan are for profit, this study bases on private providers who are considering opening a senior daycare center and are looking for a location for providing the services. The network that consists of a control network and four merit sub-networks is constructed, as shown in Figure 3.

There are three strategic criteria, namely, economics, politics, and social. The *economics* strategic criterion considers the finance of the center, the income level of the neighborhood, and the optimization of the resources. The *politics* strategic criterion considers government regulations and political trend for long-term care. The *social* strategic criterion considers human capital, social responsibility of the center, and social trend for long-term care. There are criteria under each of the merits. The definitions of the criteria are shown in Table 3. Four daycare center locations, *A*_1_, *A*_2_, *A*_3_ and *A*_4_, in Taiwan are evaluated. Experts in the field are invited to perform the evaluations in the case study. There are five experts, including two potential senior daycare center entrepreneurs who are considering opening a senior daycare center, two consultants in the health care industry, and one scholar in the senior care field.

*Phase I: Calculate priorities of BOCR merits*

Experts are invited to fill out the questionnaire for evaluating the importance of the strategic criteria. Each expert pairwise compares the three strategic criteria by using the nine different linguistic terms shown in Table 1, and a fuzzy aggregated pairwise comparison matrix is prepared next. For example, the pairwise comparison between *S*_1_ and *S*_2_ by the experts are “very intermediate important,” “moderately important,” “equally important,” “very intermediate important,” and “equally important.” The fuzzy numbers are (1, 2, 3), (2, 3, 4), (1, 1, 1), (1, 2, 3), and (1,1,1). The fuzzy aggregated number is $(1.149,\;1.644,\;2.048)\;=\;((1\times 2\times 1\times 1\times 1{)}^{1/5},\;(2\times 3\times 1\times 2\times 1{)}^{1/5},{(3\times 4\times 1\times 3\times 1)}^{1/5})$. The fuzzy aggregated pairwise comparison matrix for the strategic criteria is:

$$\begin{array}{c} \begin{array}{ccc} \;\;\;\;S_{1} & \;\;\;\;\;\;\;S_{2} & \;\;\;\;\;\;\;S_{3} \end{array}\\ \begin{array}{l} {\tilde{A}}_{S}=\begin{array}{c} S_{1}\\ S_{2}\\ S_{3} \end{array}\left[\begin{array}{ccc} \left(1,\;1,\;1\right) & \left(1.149,\;1.644,\;2.048\right) & \left(1.149,\;1.431,\;1.644\right)\\ {\left(1.149,\;1.644,\;2.048\right)}^{-1} & \left(1,\;1,\;1\right) & \left(1.000,\;1.320,\;1.552\right)\\ {\left(1.149,\;1.431,\;1.644\right)}^{-1} & {\left(1.000,\;1.320,\;1.552\right)}^{-1} & \left(1,\;1,\;1\right) \end{array}\right] \end{array} \end{array}$$

The centroid method is adopted next to prepare a defuzzified comparison matrix. For example, the fuzzy aggregated number for the comparison between *S*_1_ and *S*_2_ is (1.149, 1.644, 2.048), the defuzzified comparison between *S*_1_ and *S*_2_ is 1.613. The defuzzified aggregated pairwise comparison matrix for the strategic criteria is:

$$\begin{array}{c} \begin{array}{ccc} \;\;\;\;S_{1} & \;S_{2} & \;S_{3} \end{array}\\ \begin{array}{l} A_{S}=\begin{array}{c} S_{1}\\ S_{2}\\ S_{3} \end{array}\left[\begin{array}{ccc} 1.000 & 1.613 & 1.408\\ 0.620 & 1.000 & 1.290\\ 0.710 & 0.775 & 1.000 \end{array}\right] \end{array} \end{array}$$

The priority vector and $\lambda_{\max}$ of the defuzzified-aggregated pairwise comparison matrix for the strategic criteria are obtained:

$$w_{s}=\begin{array}{c} S_{1}\\ S_{2}\\ S_{2} \end{array}\left[\begin{array}{c} 0.429\\ 0.303\\ 0.268 \end{array}\right],\;\lambda_{\max}=3.062.$$

Next, the importance of each merit to each strategic criterion is calculated. The experts are asked to evaluate the importance of each merit (B, O, C, R) with respect to each strategic criterion using a five-level linguistic scale, and a triangular fuzzy number to represent the opinion: very high (7,9,9), high (5,7,9), medium (3,5,7), low (1,3,5), and very low (1,1,3). Geometric mean approach is adopted to aggregate experts’ opinions, and the aggregated fuzzy weight of each merit with respect to each strategic criterion is shown in Table 4. The centroid method is used to defuzzify the fuzzy numbers, as shown in Table 5. Based on the priorities of strategic criteria and the crisp weights of the four merits, the overall priorities of the four merits are calculated, as shown in Table 5. The normalized priorities of the four merits are calculated, as shown in the last column of Table 5: benefits (*b*), 0.32569; opportunities (*o*), 0.25318; costs (*c*), 0.21189; and risks (*r*), 0.20925.

*Phase II: Determine interrelationships among criteria under each merit*

The FISM is adopted to determine the interrelationship among the criteria under the same upper-level merit. Experts are invited to evaluate the interrelationships between each two criteria under each merit using the five linguistic variables listed in Table 2. The linguistic variables are transformed into triangular fuzzy numbers using Table 2, and the geometric mean approach is applied to synthesize experts’ responses. The aggregated fuzzy relation matrix for each merit can be formed. For example, fuzzy relation matrix for the criteria under the opportunities merit, $D_{o}^{\alpha }$, is:

$$D_{o}^{\alpha }=\left[\begin{array}{cccc} 0 & \left[0.41_{}^{\alpha },0.69_{}^{\alpha }\right] & \left[0.34_{}^{\alpha },0.64_{}^{\alpha }\right] & \left[0.49_{}^{\alpha },0.72_{}^{\alpha }\right]\\ \left[0.18_{}^{\alpha },0.46_{}^{\alpha }\right] & 0 & \left[0.41_{}^{\alpha },0.67_{}^{\alpha }\right] & \left[0.71_{}^{\alpha },0.92_{}^{\alpha }\right]\\ \left[0.20_{}^{\alpha },0.49_{}^{\alpha }\right] & \left[0.25_{}^{\alpha },0.50_{}^{\alpha }\right] & 0 & \left[0.56_{}^{\alpha },0.82_{}^{\alpha }\right]\\ \left[0.28_{}^{\alpha },0.56_{}^{\alpha }\right] & \left[0.25_{}^{\alpha },0.54_{}^{\alpha }\right] & \left[0.37_{}^{\alpha },0.66_{}^{\alpha }\right] & 0 \end{array}\right].$$

The defuzzified aggregated relation matrix for each merit is prepared by adopting α -cuts and the degree of satisfaction of the experts on judgment. Let α=0.5,β=0.6, the defuzzified aggregated relation matrix for the opportunities merit, $D_{o}^{0.5}$, is:

$$D_{o}^{0.5}=\left[\begin{array}{cccc} 0 & 0.57 & 0.52 & 0.64\\ 0.35 & 0 & 0.57 & 0.84\\ 0.37 & 0.40 & 0 & 0.72\\ 0.45 & 0.42 & 0.54 & 0 \end{array}\right]$$

A threshold value of 0.50 is set to determine whether there is a significant relation between each two criteria, and binary relation matrix for each merit is obtained. The binary relation matrix for the opportunities merit is:

$$\begin{array}{c} \begin{array}{cccc} \;\;\;o_{1} & o_{2} & o_{3} & o_{3} \end{array}\\ \begin{array}{l} D_{O}=\begin{array}{c} o_{1}\\ o_{2}\\ o_{3}\\ o_{3} \end{array}\left[\begin{array}{cccc} \;0 & \;1 & \;1 & \;1\\ \;0 & \;0 & \;1 & \;1\\ \;0 & \;0 & \;0 & \;1\\ \;0 & \;0 & \;1 & \;0 \end{array}\right] \end{array} \end{array}$$

The initial reachability matrix for each merit is calculated. For example, the initial reachability matrix for the opportunities merit is:

$$\begin{array}{l} H_{O}=D_{O}+I=\;\left[\begin{array}{cccc} 0 & 1 & 1 & 1\\ 0 & 0 & 1 & 1\\ 0 & 0 & 0 & 1\\ 0 & 0 & 1 & 0 \end{array}\right]+\left[\begin{array}{cccc} 1 & 0 & 0 & 0\\ 0 & 1 & 0 & 0\\ 0 & 0 & 1 & 0\\ 0 & 0 & 0 & 1 \end{array}\right]=\left[\begin{array}{cccc} 1 & 1 & 1 & 1\\ 0 & 1 & 1 & 1\\ 0 & 0 & 1 & 1\\ 0 & 0 & 1 & 1 \end{array}\right]\\ {H_{O}}^{*}=\left[\begin{array}{cccc} 1 & 1 & 1 & 1\\ 0 & 1 & 1 & 1\\ 0 & 0 & 1 & 1\\ 0 & 0 & 1 & 1 \end{array}\right] \end{array}$$

**Figure 4:**
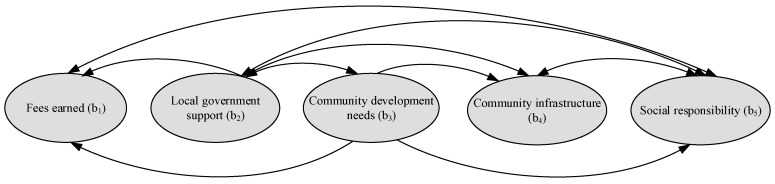
Sub-network for the benefits merit.

**Figure 5:**
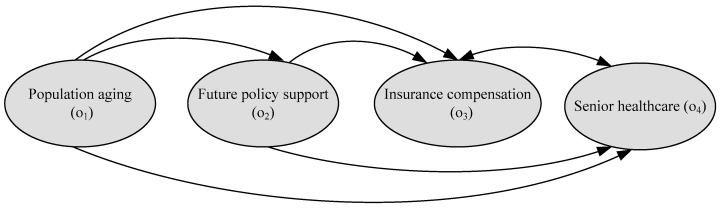
Sub-network for the opportunities merit.

**Figure 6:**
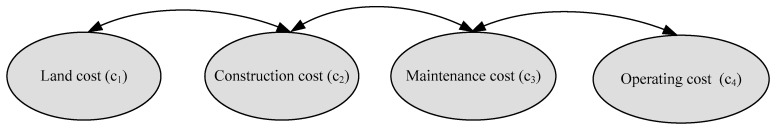
Sub-network for the costs merit.

**Figure 7:**
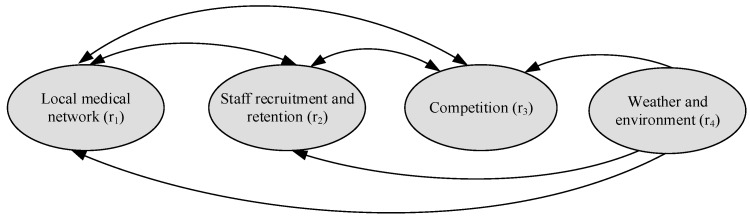
Sub-network for the risks merit.

The interrelationship among the criteria under each merit can be drawn; for example, based on $H_{O}^{*}$, the interrelationship among the four criteria under the opportunities merit, i.e., a sub-network for the opportunities merit. The same procedure is used to determine the sub-network for the benefits, costs, and risks merits. The sub-networks for the benefits, opportunities, costs, and risks merits are shown in Figure 4, Figure 5, Figure 6 and Figure 7, respectively. The direction of an arrow indicates dependence, and a two-way arrow represents the interdependency between two criteria.

*Phase III: Calculate priorities of criteria and of alternatives under each merit*

Based on Figure 3 and the sub-networks for the four merits (Figure 4, Figure 5, Figure 6 and Figure 7), a pairwise comparison questionnaire is prepared. Experts are invited to pairwise compare the importance of the criteria under each merit, the interdependence among the criteria under each merit, and the expected performance of the senior daycare center location alternatives with respect to each criterion. Steps 15−16 are carried out, and the unweighted supermatrix, the weighted supermatrix, and the limit supermatrix for each merit are prepared. For example, the unweighted supermatrix, the weighted supermatrix, and the limit supermatrix for the opportunities merit are shown in Table 6, Table 7, and Table 8, respectively. The priorities of locations A_1_, A_2_, A_3_, and A_4_ are 0.29024, 0.34116, 0.20209, and 0.16651, respectively. The priorities and ranks of locations under each merit are listed in Table 9. For the benefits and opportunities merits, the priorities are larger the better. That is, a larger priority of a location indicates a higher performance of the location under the specific merit. For example, under the opportunities merit, A_2_, with the largest priority of 0.34116, ranks the first among the four location alternatives. On the other hand, for the costs and risks merits, the smaller the priorities the better. For example, under the costs merit, A_3_, with the smallest priority of 0.19783, ranks the first among the four locations. This implies that A_3_ has the lowest costs among the four locations.

*Phase IV: Calculate overall priorities of senior daycare center locations*

The overall priorities of the location alternatives can be calculated by six different methods: additive, probabilistic additive, subtractive, multiplicative priority powers, multiplicative, and bipolar. Based on the results in Table 9 and Equations (8)–(12), Table 10 is prepared first, and the overall priority of each location can be calculated. For example, the overall priority of location A_1_ using each of the first five methods is as follows:

Additive:

0.32569 × 0.28901 + 0.25318 × 0.29024 + 0.21189 × 0.19298 + 0.20925 × 0.19486 = 0.24928

Probabilistic additive:

0.32569 × 0.28901 + 0.25318 × 0.29024 + 0.21189 × (1−0.31109) + 0.20925 × (1−0.31382) = 0.45717

Subtractive:

0.32569 × 0.28901 + 0.25318 × 0.29024 - 0.21189 × 0.31109 - 0.20925 × 0.31382 = 0.03603

Multiplicative priority powers:

0.28901^0.32569^ × 0.29024^0.25318^ × 0.19298^0.21189^ × 0.19486^0.20925^ = 0.24456

Multiplicative:

0.28901 × 0.29024 / (0.31109 × 0.31382) = 0.85923

The overall priorities and rankings of the location alternatives under the first five methods are shown in Table 11. For example, under the additive method, location A_2_, with the largest priority of 0.27706, ranks the first among the four location alternatives, followed by A_3_, with a priority of 0.25716. The results show that the rankings under the five methods are the same. Therefore, location A_2_ is the most appropriate for constructing a senior daycare center.

The calculation using the bipolar method is as follows:

Selectability index

0.32569 / (0.32569 + 0.25318) × 0.28901 + 0.25318 /(0.32569 + 0.25318) × 0.29024 = 0.28955

Rejectability index

0.21189 / (0.21189 + 0.20925) × 0.31109 + 0.20925 / (0.21189 + 0.20925) × 0.31382 = 0.31244

**Figure 8:**
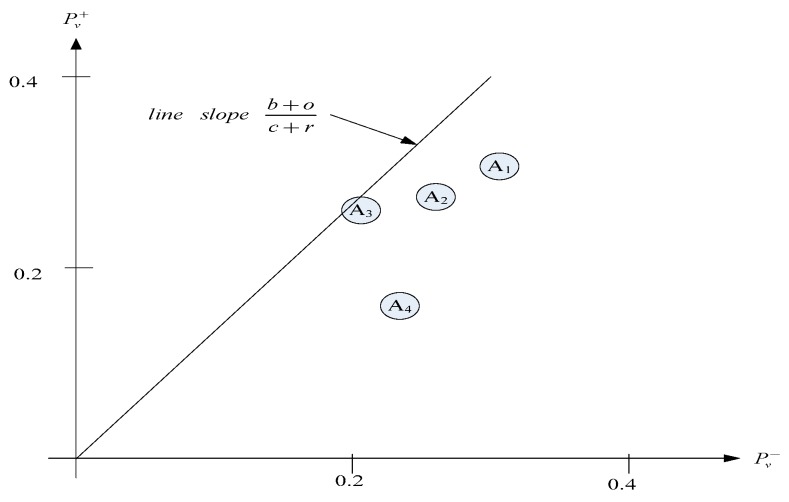
Positions of location alternatives in (rejectability, selectability) plane under bipolar method.

The calculation results are shown in Table 12. Based on the selectability measure, A_1_, with the highest value, ranks the first, followed by A_2_, then A_3_. Based on the rejectability measure, A_3_, with the lowest value, ranks the first, followed by A_4_, then A_2_. The positions of the location alternatives in (rejectability, selectability) plane can be observed in Figure 8. With the priorities of benefits (*b*), 0.32569, opportunities (*o*), 0.25318, costs (*c*), 0.21189, and risks (*r*), 0.20925, the selectability priority is 0.57887 (0.32569 + 0.25318) and the rejectability priority is 0.42114 (0.21189 + 0.20925). Thus, a line from the origin with a slope 1.37343 (0.57887/0.42114) is drawn, and alternatives that are on the line or on the top left side are preferable. Under the same $P_{v}^{-}$, the location with a higher $P_{v}^{+}$ should be selected. On the other hand, under the same $P_{v}^{+}$, the location with a lower $P_{v}^{-}$ should be selected. In this case, location A_3_, which is nearest to the line, should be selected.

**Figure 9:**
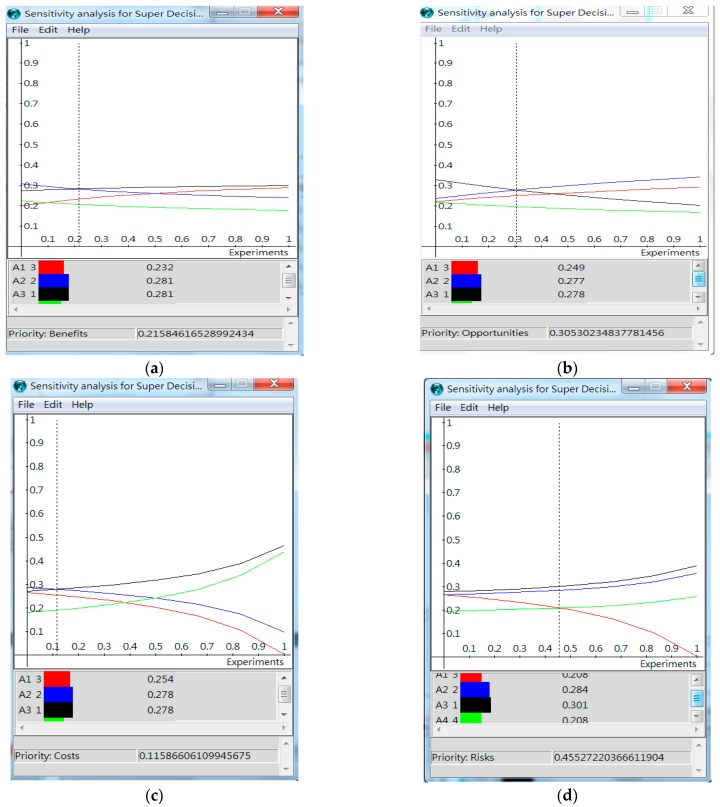
Sensitivity analysis under the additive method. (**a**) Changes in the priority of benefits; (**b**) changes in the priority of opportunities; (**c**) changes in the priority of costs; (**d**) changes in the priority of risks.

A sensitively analysis can be performed to see the robustness of the solution. For instance, the changes in the priorities and ranking of the alternatives can be examined when the priorities of the merits change. Using the additive method as an example, the results are shown in Figure 9 [24]. For example, when the priority of the benefits merit changes, the priorities and ranking of the four senior daycare center locations can be observed in Figure 9a. Under the current priority of the benefits merit, the priorities of A_1_ to A_4_ are 0.24928, 0.26517, 0.27226, and 0.21331, respectively. The ranking is A_3_, A_2_, A_1_, and A_4_. When the priority of the benefits merit decreases to 0.21585, both A_2_ and A_3_ rank the first. When the priority of the benefits merit increases to 0.49737, both A_1_ and A_3_ rank the first.

The results of the sensitivity analysis under the additive method are also shown in Table 13. Use the benefits merit as an example. The original priority of benefits (*b*) is 0.32569, we want to learn how much priority *b* needs to increase or decrease to make the best alternative change from A_3_ to another alternative. No matter how priority *b* increases, the best alternative remains to be location A_3_. However, the best alternatives become both A_3_ and A_2_ when *b* decreases to 0.21585. When the priority *b* is lower than 0.21585, the best alternative becomes A_2_. The original priority of opportunities (*o*) is 0.25318. When priority increases to 0.30530, the best alternative becomes A_2_. The original priority of costs (*c*) is 0.21189. When priority decreases to 0.11587, the best alternative becomes A_2_. The original priority of risks (*r*) is 0.20925. No matter the priority increases or decreases, the best alternative remained to be A_3_. To summarize, when priority *b* decreases by less than 33.73% ((0.21585−0.32569)/0.32568 = −33.73%), priority *o* increases by less than 20.59%, or priority *r* decreases by less than 45.32%, alternative A_3_ remains to be the best alternative. Since the above changes in the priorities of the merits are rather large, and therefore, rather unlikely, the current solution of the best alternative is quite robust.

The importance of the criteria evaluated by the experts can be observed here. The priorities of the criteria are shown in Table 14. For example, under the benefits merit, fees earned (b1) has the highest priority of 0.32443, followed by community development needs (b3) with a priority of 0.24061. Under the opportunities merit, the most important criterion is population aging (o1), with a priority of 0.37098, followed by future policy support (o2), with a priority of 0.25842. Under the costs merit, land cost (c1) has the highest priority of 0.32568, followed by construction cost (c2) with a priority of 0.28807. Under the risks merit, the most important criterion is competition (r3), with a priority of 0.42395, followed by staff recruitment and retention (r2), with a priority of 0.29666. From the global point of view, that is, considering the priorities of the merits, the top five criteria, in descending order, are fees earned (b1) (0.10566), population aging (o1) (0.09392), competition (r3) (0.08871), community development needs (b3) (0.07836), and land cost (c1) (0.06901).

## 6. Conclusions

Population aging is present in most developed countries. As the silver-haired population increases, the need of long-term care is becoming an imminent issue. Senior daycare center provides supervised care for the seniors during the day while living at home in the evenings. In addition, seniors can interact with and take care of each other in the center, and they can have socialization and mental stimulation. Family of the seniors can have a higher quality of life too, especially if family members are working.

In this study, a model is constructed for evaluating and selecting senior daycare center locations. A network with benefits, opportunities, costs, and risks (BOCR) merits is developed, and the interrelationships among the criteria under each merit are determined by adopting the fuzzy interpretative structural modeling (FISM). The fuzzy analytic network process (FANP) is applied to calculate the importance weights of the criteria under each merit, and the ranking of senior daycare center locations can be generated.

The model can be used by long-term care service providers who are making decisions on where to set up senior daycare centers. By applying the model, they can understand what important criteria should be considered when selecting an appropriate location and the ranking of the locations. With the first successful step, they can build up senior daycare centers that can provide good services to the seniors and in turn meet their own organization goals.

To the authors’ knowledge, the proposed model is the first one to be adopted to solve the senior daycare center location evaluation problem. The application in the senior daycare center location evaluation is innovative in the public health industry. In the future, different models may be proposed to solve the problem, and a comparison of models with the one in this research can be performed.

## Figures and Tables

**Table 1:** Triangular fuzzy numbers for fuzzy analytic network process (FANP).

Linguistic Variables	Positive Triangular Fuzzy Numbers	Positive Reciprocal Triangular Fuzzy Numbers
Extremely important/good	(9,9,9)	(1/9,1/9,1/9)
Extremely intermediate important/good	(7,8,9)	(1/9,1/8,1/7)
Very important/good	(6,7,8)	(1/8,1/7,1/6)
Very intermediate important/good	(5,6,7)	(1/7,1/6,1/5)
Strongly important/good	(4,5,6)	(1/6,1/5,1/4)
Strongly intermediate important/good	(3,4,5)	(1/5,1/4,1/3)
Moderately important/good	(2,3,4)	(1/4,1/3,1/2)
Moderately intermediate important/good	(1,2,3)	(1/3,1/2,1)
Equally important/good	(1,1,1)	(1,1,1)

**Table 2:** Triangular fuzzy numbers for fuzzy interpretative structural modeling (FISM).

Linguistic Variables	Triangular Fuzzy Numbers
Completely related	(0.75,1,1)
Strongly related	(0.5,0.75,1)
Fairly related	(0.25,0.5,0.75)
Low related	(0.01,0.25,0.5)
Unrelated	(0.01,0.01,0.01)

**Table 3:** Senior daycare center location evaluation criteria.

Merits	Criteria	Definition
Benefits	Fees earned (b1)	Service fee collected from seniors/families and other parties.
	Local government support (b2)	Financial and non-financial supports from local government.
	Community development needs (b3)	Long-term care service needs in the neighborhood.
	Community infrastructure (b4)	Physical structures and facilities in the surrounding environment (e.g., buildings, roads, medical institutions, and parks).
	Social responsibility (b5)	Improving the quality of life of the workforce and their families, and securing the welfare of the local community and society, etc.
Opportunities	Population aging (o1)	Senior population trend in the area.
	Future policy support (o2)	Future government funding or incentive programs to centers and/or seniors.
	Insurance compensation (o3)	Compensation for seniors to cover the long-term care service fee.
	Senior healthcare (o4)	Providing preventive healthcare and illness treatment to the participants.
Costs	Land cost (c1)	Acquisition or long-term rental cost of the land.
	Construction cost (c2)	Construction of the building and acquisition of equipment and hardware.
	Maintenance cost (c3)	Day-to-day maintenance of the center and the equipment.
	Operating cost (c4)	Cost of running the service (e.g., salaries of staff, supplies, utilities), and cost of transportation services, public transit, and family pickup/drop-off.
Risks	Local medical network (r1)	Insufficient medical institutions in the area to provide services to the participants.
	Staff recruitment and retention (r2)	Difficulties in recruiting and retaining staff (e.g., nurses, aides, rehabilitation therapist, and administration personnel) in the area.
	Competition (r3)	Other senior daycare centers or long-term care services in the neighborhood.
	Weather and environment (r4)	Temperature, humidity, wind, rain, climate changes, and ambient air quality in the area.

**Table 4:** Aggregated fuzzy weights of merits with respect to strategic criteria.

	*S*_1_^1^	*S*_2_^2^	*S*_3_^3^
Benefits	(0.758, 1.059, 1.351)	(0.699, 1.000, 1.431)	(1.217, 1.719,2.352)
Opportunities	(1.585, 2.352, 3.022)	(0.608, 0.803, 1.084)	(0.488, 0.803, 1.351)
Costs	(1.516, 2.268, 2.930)	(0.871, 1.149, 1.431)	(1.516, 2.268, 2.930)
Risks	(0.922, 1.431, 2.048)	(0.803, 1.149, 1.552)	(0.699, 0.871, 1.149)

^1^ Economics strategic criterion. ^2^ Politics strategic criterion. ^3^ Social strategic criterion.

**Table 5:** Normalized priorities of the merits (*b*, *o*, *c*, *r*).

	*S*_1_ (0.429)	*S*_2_ (0.303)	*S*_3_ (0.268)	Overall Priorities	Normalized Priorities
Benefits	2.238	1.150	2.238	1.90834	0.32569
Opportunities	2.320	0.832	0.881	1.48348	0.25318
Costs	1.056	1.043	1.763	1.24154	0.21189
Risks	1.467	1.168	0.906	1.22606	0.20925

**Table 6:** Unweighted supermatrix for the opportunities merit.

	G ^1^	o_1_^2^	o_2_^3^	o_3_^4^	o_4_^5^	A_1_^6^	A_2_^7^	A_3_^8^	A_4_^9^
g	1	0	0	0	0	0	0	0	0
o_1_	0.37098	1	0.58159	0.2828	0.38967	0	0	0	0
o_2_	0.25842	0	0.41841	0.28818	0.24034	0	0	0	0
o_3_	0.20206	0	0	0.21473	0.18889	0	0	0	0
o_4_	0.16853	0	0	0.21429	0.1811	0	0	0	0
A_1_	0	0.25533	0.38571	0.305	0.27714	1	0	0	0
A_2_	0	0.33468	0.29218	0.39436	0.40596	0	1	0	0
A_3_	0	0.23076	0.16557	0.15752	0.17154	0	0	1	0
A_4_	0	0.17923	0.15654	0.14312	0.14536	0	0	0	1

^1^ Goal. ^2^ Population aging. ^3^ Future policy support. ^4^ Insurance compensation. ^5^ Senior healthcare. ^6^ Location 1. ^7^ Location 2. ^8^ Location 3. ^9^ Location 4.

**Table 7:** Weighted supermatrix for the opportunities merit.

	g	o_1_	o_2_	o_3_	o_4_	A_1_	A_2_	A_3_	A_4_
g	0.5	0	0	0	0	0	0	0	0
o_1_	0.18549	0.5	0.2908	0.1414	0.19484	0	0	0	0
o_2_	0.12921	0	0.20921	0.14409	0.12017	0	0	0	0
o_3_	0.10103	0	0	0.10736	0.09445	0	0	0	0
o_4_	0.08427	0	0	0.10715	0.09055	0	0	0	0
A_1_	0	0.12766	0.19286	0.1525	0.13857	1	0	0	0
A_2_	0	0.16734	0.14609	0.19718	0.20298	0	1	0	0
A_3_	0	0.11538	0.08278	0.07876	0.08577	0	0	1	0
A_4_	0	0.08962	0.07827	0.07156	0.07268	0	0	0	1

**Table 8:** Limit supermatrix for the opportunities merit.

	g	o_1_	o_2_	o_3_	o_4_	A_1_	A_2_	A_3_	A_4_
g	0	0	0	0	0	0	0	0	0
o_1_	0	0	0	0	0	0	0	0	0
o_2_	0	0	0	0	0	0	0	0	0
o_3_	0	0	0	0	0	0	0	0	0
o_4_	0	0	0	0	0	0	0	0	0
A_1_	0.29024	0.25533	0.33777	0.29976	0.28283	1	0	0	0
A_2_	0.34116	0.33468	0.30781	0.36847	0.37383	0	1	0	0
A_3_	0.20209	0.23076	0.18954	0.17786	0.18726	0	0	1	0
A_4_	0.16651	0.17923	0.16489	0.15391	0.15609	0	0	0	1

**Table 9:** Priorities and ranks of locations under each merit.

Merits	Benefits		Opportunities		Costs		Risks	
alternatives	priority	rank	priority	rank	priority	rank	priority	rank
A_1_	0.28901	2	0.29024	2	0.31109	4	0.31382	4
A_2_	0.23688	3	0.34116	1	0.28732	3	0.22305	2
A_3_	0.29840	1	0.20209	3	0.19783	1	0.21467	1
A_4_	0.17571	4	0.16651	4	0.20377	2	0.24847	3

**Table 10:** Calculation of the priorities of locations under each merit.

Merits	Benefits	Opportunities	Costs			Risks		
priorities	0.32569	0.25318	0.21189			0.20925		
alternatives	normalized	normalized	normalized	reciprocal	normalized	normalized	reciprocal	normalized
					reciprocal			reciprocal
A_1_	0.28901	0.29024	0.31109	3.21450	0.19298	0.31382	3.18657	0.19486
A_2_	0.23688	0.34116	0.28732	3.48044	0.20894	0.22305	4.48334	0.27416
A_3_	0.29840	0.20209	0.19783	5.05485	0.30346	0.21467	4.65836	0.28486
A_4_	0.17571	0.16651	0.20377	4.90749	0.29462	0.24847	4.02467	0.24611

**Table 11:** Overall priorities of locations.

Methods	Additive		Probabilistic Additive		Subtractive		Multiplicative Priority Powers		Multiplicative	
alternatives	priority	rank	priority	rank	priority	rank	priority	rank	priority	rank
A_1_	0.24928	3	0.45717	3	0.03603	3	0.24456	3	0.85923	3
A_2_	0.26517	2	0.47711	2	0.05597	2	0.26084	2	1.26102	2
A_3_	0.27226	1	0.48265	1	0.06151	1	0.26870	1	1.41999	1
A_4_	0.21331	4	0.42536	4	0.00422	4	0.20752	4	0.57787	4

**Table 12:** Bipolar priorities of locations.

	Bipolar Selectability		Bipolar Rejectability	
alternatives	priority	rank	priority	rank
A_1_	0.28955	1	0.31244	4
A_2_	0.28249	2	0.25539	3
A_3_	0.25628	3	0.20620	1
A_4_	0.17169	4	0.22598	2

**Table 13:** Sensitivity analysis under different priorities of merits under the additive method.

Merits	Priority Changes	Alternative Ranking
Benefits	0≤b<0.09745	$A_{1}<A_{4}<A_{3}<A_{2}$
	b=0.09745	$A_{1}=A_{4}<A_{3}<A_{2}$
	0.09745<b<0.21585	$A_{4}<A_{1}<A_{3}<A_{2}$
	b=0.21585	$A_{4}<A_{1}<A_{3}=A_{2}$
	0.21585<b<0.49737	$A_{4}<A_{1}<A_{2}<A_{3}$
	0.49737=b	$A_{4}<A_{1}=A_{2}<A_{3}$
	0.49737<b≤1	$A_{4}<A_{2}<A_{1}<A_{3}$
Opportunities	0≤o<0.30530	$A_{4}<A_{1}<A_{2}<A_{3}$
	0=0.30530	$A_{4}<A_{1}<A_{2}=A_{3}$
	0.30530<o<0.44737	$A_{4}<A_{1}<A_{3}<A_{2}$
	o=0.44737	$A_{4}<A_{1}=A_{3}<A_{2}$
Costs	0≤c<0.11587	$A_{4}<A_{1}<A_{3}<A_{2}$
	c=0.11587	$A_{4}<A_{1}<A_{3}=A_{2}$
	0.11587<c<0.37897	$A_{4}<A_{1}<A_{2}<A_{3}$
	c=0.37897	$A_{4}=A_{1}<A_{2}<A_{3}$
	0.37897<c<0.48948	$A_{1}<A_{4}<A_{2}<A_{3}$
	0.48948=c	$A_{1}<A_{4}=A_{2}<A_{3}$
	0.48948<c≤1	$A_{1}<A_{2}<A_{4}<A_{3}$
Risks	0≤r<0.01326	$A_{4}<A_{2}<A_{1}<A_{3}$
	0=0.01326	$A_{4}<A_{2}=A_{1}<A_{3}$
	0.01326<r<0.45527	$A_{4}<A_{1}<A_{2}<A_{3}$
	r=0.45527	$A_{4}=A_{1}<A_{2}<A_{3}$
	0.45527<r≤1	$A_{1}<A_{4}<A_{2}<A_{3}$

**Table 14:** Priorities of criteria.

Merits	Criteria	Local Priorities	Local Rank	Global Priorities	Global Rank
Benefits (0.32569)	Fees earned (b_1_)	0.32443	1	0.10566	1
	Local government support (b_2_)	0.20222	3	0.06586	6
	Community development needs (b_3_)	0.24061	2	0.07836	4
	Community infrastructure (b_4_)	0.12767	4	0.04158	13
	Social responsibility (b_5_)	0.10506	5	0.03422	16
Opportunities (0.25318)	Population aging (o_1_)	0.37098	1	0.09392	2
	Future policy support (o_2_)	0.25842	2	0.06543	7
	Insurance compensation (o_3_)	0.20206	3	0.05116	10
	Senior healthcare (o_4_)	0.16853	4	0.04267	12
Costs (0.21189)	Land cost (c_1_)	0.32568	1	0.06901	5
	Construction cost (c_2_)	0.28807	2	0.06104	9
	Maintenance cost (c_3_)	0.16287	4	0.03451	15
	Operating cost (c_4_)	0.22338	3	0.04733	11
Risks (0.20925)	Local medical network (r_1_)	0.18823	3	0.03939	14
	Staff recruitment and retention (r_2_)	0.29666	2	0.06208	8
	Competition (r_3_)	0.42395	1	0.08871	3
	Weather and environment (r_4_)	0.09117	4	0.01908	17
